# The Impact of Recovery Practices Adopted by Professional Tennis Players on Fatigue Markers According to Training Type Clusters

**DOI:** 10.3389/fspor.2020.00109

**Published:** 2020-09-02

**Authors:** Mathilde Poignard, Gaël Guilhem, Quentin de Larochelambert, Bernard Montalvan, François Bieuzen

**Affiliations:** ^1^French Institute of Sport (INSEP), Laboratory Sport, Expertise and Performance (EA 7370), Paris, France; ^2^French Tennis Federation, Paris, France; ^3^IRMES (Institut de Recherche bioMedicale et d'Epidemiologie du Sport), INSEP, Paris, France; ^4^Institut National du Sport du Québec, Montréal, QC, Canada

**Keywords:** training load, recovery practices, cryotherapy, cooling strategies, muscle soreness, perceived fatigue

## Abstract

**Introduction:** Modern tennis players face congested schedules that force the adoption of various recovery strategies. Thus, recovery must be fine-tuned with an accurate quantification of its impacts, especially with regards to training-induced fatigue. The present study aimed to examine the training type clusters and recovery practices adopted by elite tennis players under ecological training conditions. The respective impacts of training type clusters and recovery techniques on subjective variables, which reflect the players' recovery perceptions, were subsequently determined.

**Methods:** During 15 consecutive months, a total of 35 elite tennis players filled out questionnaires to report their daily training load, training session content, adopted recovery modalities after training, and perceived recovery.

**Results:** The hierarchical analysis identified three clusters: “combined tennis and S&C training,” “predominant tennis training” and “predominant S&C training.” *Muscle soreness* and *perceived fatigue* were not significantly different among these three clusters (*p* = 0.07–0.65). Across the 146 recorded training and recovery sessions, players primarily employed a combination of 2 or 3 modalities, with cooling strategies being the most widely used technique (87.6%). Mixed linear models revealed that independent of training clusters, cooling strategies significantly reduced muscle soreness (Δ*muscle soreness*: β = −1.00, *p* = 0.02). Among the cooling techniques used, whole-body cryotherapy induced a greater perceived recovery than cold-water immersion (*p* = 0.02).

**Conclusion:** These results showed that perceived recovery was not sensitive to training clusters or the associated acute training load. However, cooling strategies were relevant for the alleviation of tennis training-induced soreness. This study represents an initial step toward a periodized approach of recovery interventions, based on the interactions between training load, training contents, and perceived recovery.

## Introduction

Elite tennis players face a continuous increase in competition density, resulting in increased physical demands and injury rates (Fu et al., 2018). To be well-prepared, players begin their training seasons around the middle of November, with a preparatory phase lasting between 5 and 7 weeks. Then, players alternate between pre-competitive and competitive phases with a training vs. competition ratio around 40–60% (Kovacs, 2018). However, these training phases are crucial, not only for fitness training but also for the development of technical and tactical skills. Composite training, combined with a congested schedule, can result in several states of fatigue, requiring coaches and athletes to implement appropriate recovery periods and techniques. Therefore, understanding (i) the nature of fatigue induced by this style of training and (ii) the recovery status of elite tennis players is necessary to optimize the periodization of appropriate recovery techniques (Kellmann et al., 2018).

Tennis-induced fatigue is the consequence of numerous factors, such as playing style, gender, training status, age, playing surface, ball type, and environment (Fernandez-Fernandez et al., 2009), which result in various physiological and psychological disturbances. These potential stressors can be evaluated through training/competition load indicators designed to assess whether an athlete is adapting well to the training, competition loads, and stimuli. The Session-Rating of Perceived Exertion (sRPE) is an ecological and non-invasive training/competition load indicator which has been validated for various sports, including tennis (Foster et al., 2001; Gomes et al., 2015; Haddad et al., 2017). Recent studies have evaluated the loads imposed during tennis competitions (Ojala and Hakkinen, 2013; Duffield et al., 2014), but few studies have addressed the daily or weekly distribution of training sessions and training loads imposed on an elite tennis player under ecological conditions (Murphy et al., 2015; Vescovi, 2017).

To monitor an athlete's recovery status and to measure how the recovery modality affects post-exercise recovery, the Hooper questionnaire (Hooper and Mackinnon, 1995), which is based on multiple subjective variables, has been widely used in several studies (Bleakley et al., 2012; Bieuzen et al., 2013; Duffield et al., 2014; Costello et al., 2015; Schaal et al., 2015). Subjective variables have also recently been shown to be sensitive to changes in training loads during applied professional team sports research (Moalla et al., 2016; Thorpe et al., 2017). However, to our knowledge, no study has assessed the impacts of training loads on subjective recovery in high-level tennis players.

Investigating the effects of professional training load and recovery modalities are paramount, given that a wide variety of recovery techniques (e.g., water immersions, active recovery, stretching, whole-body cryotherapy, compression garments…) are available to tennis players. However, inconsistent results have been reported regarding the impacts of different recovery techniques on the fatigue induced by training or competition (Bahnert et al., 2013; Halson et al., 2014; Roberts et al., 2015; Dupuy et al., 2018; Tavares et al., 2019). Elite tennis centers have developed some practical guides regarding recovery techniques that are provided to coaches and athletes; however, no systematic evidence has been reported regarding the efficiencies of these techniques. Recently, a study reported that 80% of competitive tennis players adopted multiple post-exercise recovery strategies, primarily foam rolling, cold-water immersion, hot-water immersion, and the intake of protein shakes (Fleming et al., 2018). However, research examining the effects of these techniques on tennis players remains limited. For example, only one study found that combining cold water immersion with compression garments was able to alleviate post-training muscle soreness (Duffield et al., 2014). Some recent studies have improved the understanding of recovery for specific disciplines, including professional football and rugby, and have promoted recommendations for specific recovery strategies that should be applied to the highest-level athletes, based on the specific demands of these sports (Nédélec et al., 2015; Tavares et al., 2017). Thus, a better understanding of the efficiencies of the post-exercise recovery routines adopted by tennis players could help fill the gap between scientific evidence and actual practice.

In this context, the aims of the study were as follows: (i) to constitute groups according to training contents and training loads and to analyze its effects on subjective variables, used to represent perceived recovery; and (ii) to provide an overview of the recovery habits adopted by elite tennis players and to determine their effects on subjective variables, according to the defined training groups. To address these two aims, we used a hierarchical clustering approach to gather the entire dataset of training sessions into subgroups according to training content, duration, and load. This approach allowed the inclusion of the uniform and consistent categorical variable of “training” into a linear mixed model, to evaluate the impacts of recovery modalities on subjective recovery variables. We hypothesized that different clusters would elicit significantly different effects on perceived fatigue and muscle soreness (Moalla et al., 2016). Based on previous literature, we consequently expected that cold recovery interventions would have larger impacts on muscle soreness than other interventions.

## Methods

### Participants

Sixteen male players from the Association of Tennis Players (age = 19.0 ± 3.0 years; stature: 185.5 ± 7.8 cm; body mass: 77.8 ± 10.1 kg; years on circuit = 4.5 ± 5.0), sixteen female players from the Women's Tennis Association (age = 20.1 ± 4.3 years; stature: 171.6 ± 5.5 cm; body mass: 60.5 ± 4.0 kg; years on circuit = 3.7 ± 4.0), and three female junior players from the International Tennis Federation engaged in Junior Grand Slam (age = 16.0 ± 0.7 years; stature: 171.0 ± 5.0 cm; body mass: 60.0 ± 5.0 kg), were included in this study. No male junior players were included in this study. At the time of the experiment, the male players were ranked (median over the 15 months of the experiment) as follows: 16 players were in the top 1,000, including 12 in the top 500. Female players were ranked as follows: 16 players were in the top 1,000, including 9 in the top 500, and 3 with no professional ranking. Players and their parents (for minors) were informed of the procedures before they provide their written informed consent. All procedures conformed to the standards of the Declaration of Helsinki, and the study was approved by the ethics committee.

### Procedure

The training load, subjective variables, and recovery techniques were monitored, using an application designed specifically for this study. For each training day, players filled out a training load questionnaire, a recovery modalities form, and a psychometric questionnaire. Players reported the contents, duration, and intensity of both morning and afternoon training sessions. At the end of the day, before recovery (PRE) and strategies being implemented, participants indicated all of the recovery modalities that they were planning to use (from 1 to 5 recovery interventions) and filled out the psychometric questionnaire. The next morning (12–16 h after recovery, POST), before training, players filled out the same psychometric questionnaire to isolate the potential effects of the recovery modality on the subjective variables (Figure 2). All sessions performed by the participants were recorded over 15 consecutive months, only in the presence of the same investigator at the training center (201 days over the 15 months). No training sessions were recorded during the competitive phase. Prior to the study, all players were familiarized with all questionnaires included in the application.

#### Training Monitoring

The training content was considered to reflect “tennis training” when players trained on a tennis court. These training sessions included technical and tactical drills, services, point play, and non-official match play, which developed technical and tactical skills. These workouts elicited specific motor tasks associated with tennis practice, including lateral sprints, rushes, cutting-maneuvers, smashes, drop landing, and jumps. “Strength and conditioning training” (S&C) corresponded to all off-court training session, for which the primary objective was developing physical fitness specific to tennis, including aerobic exercise (high-volume, low-intensity work), anaerobic exercise (interval training, using tennis-specific work/rest intervals), speed and power training (sprinting and explosive exercises), strength training (high-repetition, low-resistance exercise), and plyometric training. Within 30 min following morning and afternoon training sessions, players indicated their rate of perceived exertion (RPE) on a ten-point category-ratio scale (CR-10 Borg Scale) modified by Foster et al. (2001). We then assessed sRPE training load for the morning and afternoon training sessions, using the methods described by Foster et al. (2001). Total sRPE training load was calculated as the sum of morning and afternoon sRPE training load values.

#### Psychometric Questionnaire

The psychometric questionnaire was adapted from the Hooper questionnaire (Hooper and Mackinnon, 1995). Immediately after training, and just before recovery, players were asked to score the 3 following subjective variables: *muscle soreness, stress*, and *perceived fatigue*. The next morning, before training, players scored the same 3 variables and 2 additional factors: *sleep quality* and *perceived recovery*. All 5 variables were presented and rated on a 0–10 cm visual analog scale (VAS), with 0.1-cm accuracy. Changes between PRE and POST measurements for *muscle soreness, perceived fatigue*, and *stress* were calculated. To minimize bias, only one investigator collected all data, to preserve consistency and homogeneity. Before the study, all players were first educated regarding the meanings of the self-reported items, according to the definitions described by the Hooper questionnaire (Hooper et al., 1995). Each tennis player was blinded to the results of the other participants. Qualitative indicators used to assist players with reporting perceptions in the psychometric questionnaire were as follows:

- *Muscle soreness: 0* = *no muscle soreness to 10* = *Very high muscle soreness*
- *Perceived fatigue: 0* = *no perceived fatigue to 10* = *extremely exhausted*
- *Stress: 0* = *no stress to 10* = *extremely stressed*
- *Sleep quality: 0* = *excellent to 10* = *very bad, with insomnia*
- *Perceived recovery: 0* = *No at all to 10* = *Completely recovered*.

#### Recovery Modalities

A total of 15 different recovery modalities were implemented by players and were pooled into 5 distinct categories. The recovery modalities that aim to decrease muscle temperature were considered to be “Cooling strategies,” including whole-body cryotherapy (WBC) (3 min at −110°C), cold-water immersion (CWI) (11 min at 11°C), and contrast water therapy (CWT) (7 repetitions of 1 min/1 min at 11/40°C). Hot-water immersion and steam room modalities were classified as “heating strategies.” We pooled foam-rolling and stretching into a “Flexibility techniques” category, as these techniques are known to improve the range of motion during passive conditions (Sands et al., 2013; Macdonald et al., 2014). Active recovery, electrostimulation, thermoneutral water immersion, compression garments (Agu et al., 1999; Menetrier et al., 2015), and external pneumatic compression were categorized as “lower limb blood flow stimulation.” We classified all therapeutic procedures that required the use of physical agents (physiotherapists and osteopaths) into the group “Physiotherapy techniques,” including joint mobilization, massages, and osteopathy.

### Data Collection and Selection

**Figure 1:**
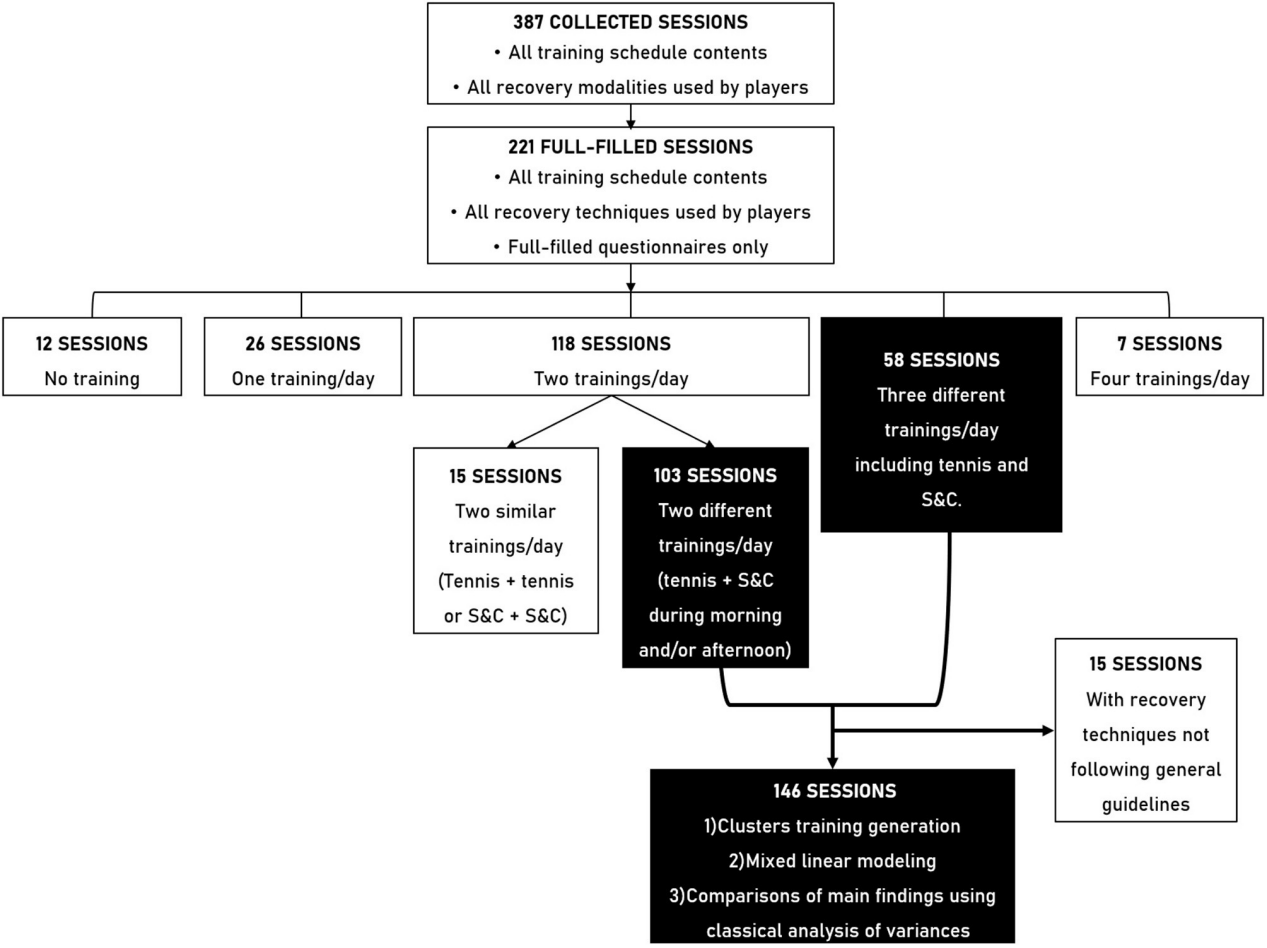
Selection of sessions flow chart. White boxes represent excluded sessions and the corresponding exclusion criteria while black boxes represent selected sessions and the corresponding inclusion criteria.

**Figure 2:**
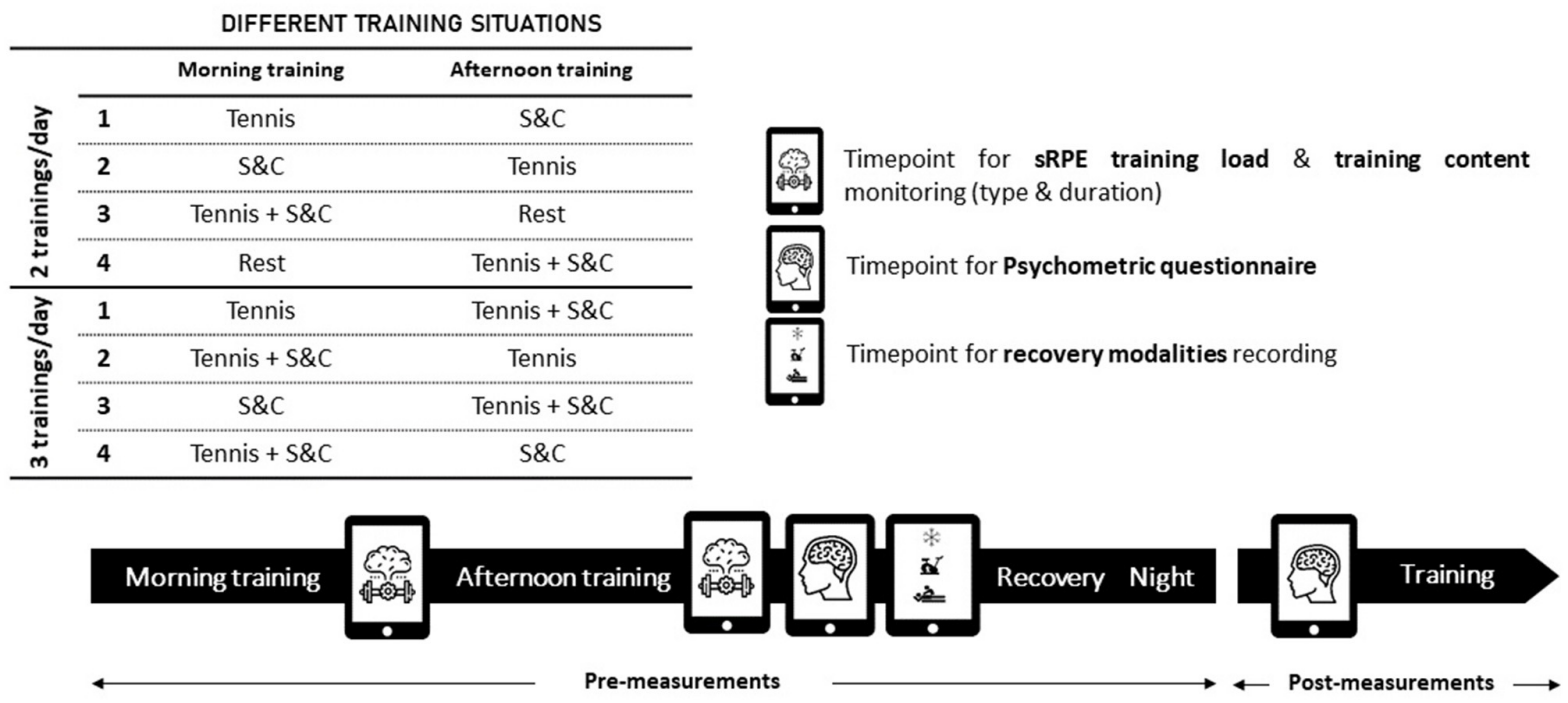
Overview of the experimental design. Tennis, tennis training; S&C, strength and conditioning training; Rest, no training. Pre-measurement, at the end of the training day; Post-measurement: the next morning, after recovery and before training.

A total of 146 sessions corresponding to the predominant training situations performed by tennis players, were selected and analyzed over the 387 sessions recorded (Figures 1, 2) in order to preserve the homogeneity of the data. These training situations corresponded to two or three training sessions per day and included at least one S&C training session and one tennis training session. Sessions that included no training, two similar training sessions (e.g., two tennis training) or four training sessions in a day were excluded from our analysis (Figure 1). Sessions that included a recovery technique which did not follow general guidelines were also excluded from further analysis. A total of 91% of the monitored sessions were recorded during a preparatory phase and 9% of the monitored sessions during a pre-competitive phase. Players filled an average of 4.1 ± 3.5 questionnaires over the 146 sessions.

### Statistical Analysis

All variables are presented as the mean ± standard deviations (SD). To divide the 146 training sessions into training profile clusters, we used principal component analysis, followed by the application of hierarchical clustering for the principal components, using *FactoMineR* (version 1.41) in R Studio (Version 1.1.463). The selection variables were total sRPE training load, total tennis training session duration, and total strength & conditioning training session duration. The squared Euclidean distances technique was used, and we fixed the possible number of clusters between 2 and 5. To investigate how training profile clusters and recovery modalities affected subjective variables, we employed linear mixed-effects models, which are an extension of linear regressions, to consider the repeated measurements within participants (146 recorded sessions from 35 players). We used the *lmer* function of the lme4 (version 1.1-21) package in R Studio, where output subjective variables (Δ*muscle soreness*, Δ*perceived fatigue, sleep quality*, and *perceived recovery*) were analyzed into separate models. We included a per player random intercept and a fixed effect for the input variables (clusters and recovery modalities). Interactions between the clusters and recovery modalities were tested, but interactions were not examined between recovery modalities, due to an insufficient number of observations. *P*-values were obtained using Welch-Satterthwaite *t*-tests, for all full models, and the significance level was fixed at *p* < 0.05. If an association between an input variable and an output subjective variable was observed, we performed an additional test [Likelihood Ratio Test (LRT)], using an analysis of variance (ANOVA) to compare a model without the input variable against a model with the input variable. All assumptions (linearity, absence of collinearity, independence, and normality of residuals) were checked with the *plot, qqnorm* function of the car package (3.0–2) in R. When a significant effect was observed in the linear mixed-effects models, differences between modalities within the same recovery category were assessed, using a One-Way ANOVA for normally distributed data or a Kruskal-Wallis test for non-normally distributed data. Similar statistical analyses were performed to determine differences between clusters for the subjective variables. When a significant main effect or interaction was observed, a Bonferroni *post-hoc* test was used to locate the difference. The level of significance was set to *p* < 0.05. These latter statistical analyses were conducted using the IBM Statistical Package for the Social Sciences (SPSS) (IBM SPSS Statistics, 20.0.0, SPSS Inc., USA). Effect sizes (ESs) were calculated using the following formula, for non-parametric data: r = z/√N, where refers to the z value obtained from the Mann-Whitney U test and N refers to the number of observations (Fritz et al., 2012). Interpretations were based on Cohen's formula, where: *r* = 0.2, 0.5, and 0.8 were considered to be small, medium, and large, respectively.

## Results

### Cluster Analysis

**Figure 3:**
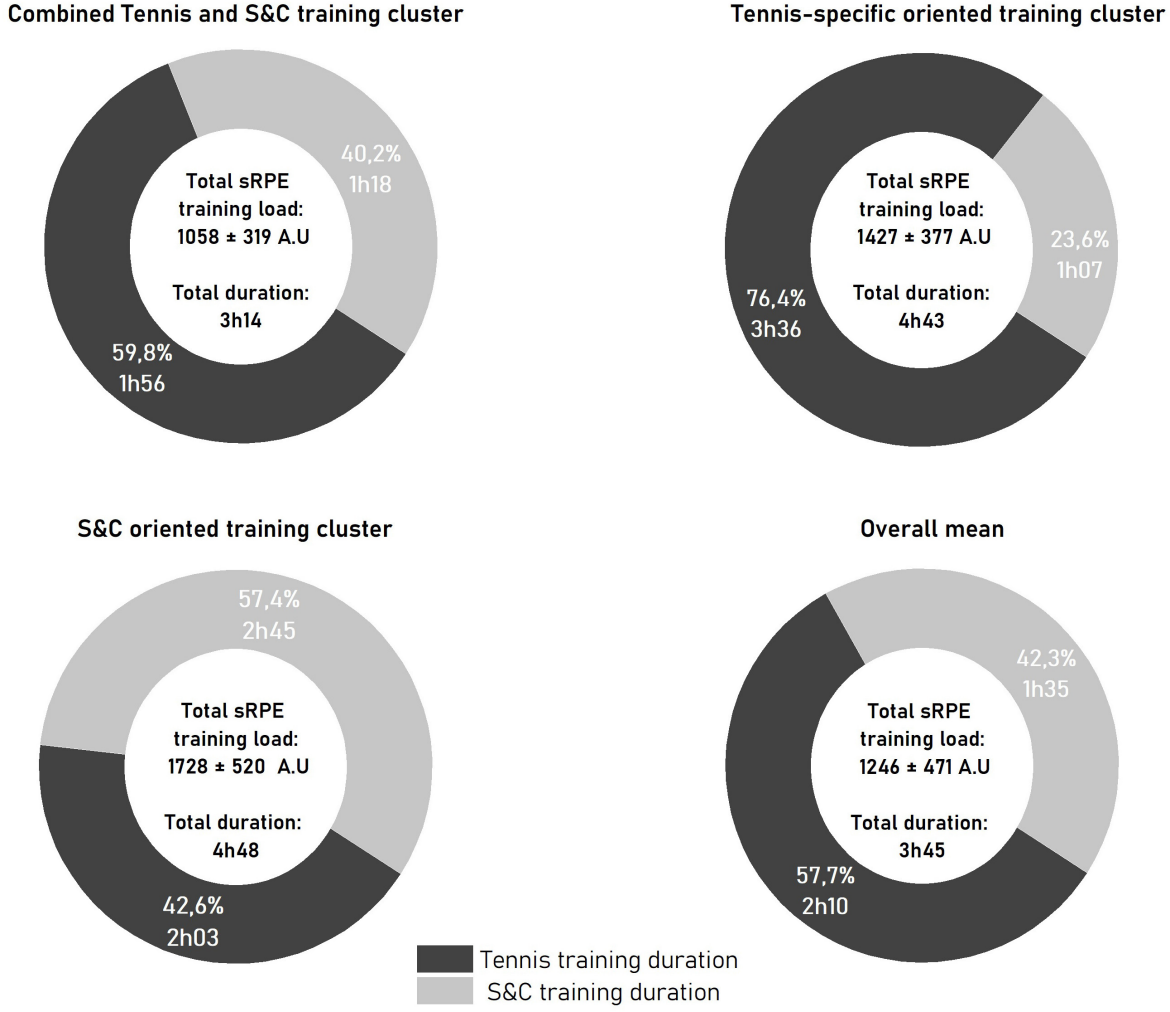
Mean ± SD of the three selections variables for each of training clusters and the overall mean. Overall mean: average of the 146 sessions; S&C (strength and conditioning) training duration expressed in hours and %; tennis training duration expressed in hours and %; total sRPE training load (total daily training load) expressed in A.U.; total duration of training in hours.

The hierarchical cluster analysis identified three training type clusters (Figure 3) which were consistent with three theorical training microcycles that are recommended during training periods. The first cluster defined as “Combined tennis and S&C training,” was statistically defined by a lower total duration of tennis training (−13.7 min), a lower total strength training duration (−17.2 min), and a smaller total sRPE training load (−187.7 A.U) compared with the means for all clusters (overall means). The second, defined as a “Tennis-specific oriented training” cluster, was statistically defined by a higher total tennis training duration (+86.1 min) and a lower total strength training duration (−28.8 min) compared with the overall means. The third, defined as a “S&C oriented training” cluster, was statistically defined by a higher total strength training duration (+70.3 min) and a higher total sRPE training load (+482.1 A.U) compared with the overall means. Kruskal-Wallis analyses showed no significant differences among clusters for *muscle soreness* (*p* = 0.10, ES: −0.19 to −0.09) and *perceived fatigue* (*p* = 0.07, ES: −0.16 to −0.08). We observed no significant differences among clusters for Δ*muscle soreness* (*p* = 0.65), Δ*perceived fatigue* (*p* = 0.98), *sleep quality* (*p* = 0.11), or *perceived recovery* (*p* = 0.12). The results for the stress subjective variables were not interpretable as a median, and the values of the lower and upper quartiles for Δ*stress* were 0 (−0.1 to 0.1) and 0.4 (0.0–1.6), respectively, for *stress* reported on PRE questionnaires.

### Recovery Techniques Adopted by Professional Tennis Players

Most of the players (69.2%) used a combination of 2 (41.8%) or 3 (27.4%) recovery modalities, with an average of 2.6 ± 1.0 techniques per session. Cooling (CWI, CWT, and WBC) was used by 87.6% of the players (Table 1). Passive mobilization was the second-most commonly used technique (61.6%), followed by physiotherapy techniques (47.9%), lower limb blood flow stimulation (46.5%), and heating strategies (14.3%).

Table 1Summary of recovery modalities used after a training day.

**Table:** 

		**Passive mobilization**	**Physiotherapy techniques**	**Heating strategies**	**Lower limb blood flow stimulation**	**Cooling strategies**
		**Number of uses per each recovery modalities**				
		Foam-rolling (n = 4); Stretching (n = 86)	Joint mobilization (n = 5); Massages (n = 53); Osteopathy (n = 12)	Hot immersion (n = 20); Steam room (n = 1)	Active recovery (n = 55); Electrostimulation (n = 2); Thermo-neutral Water Immersion (n = 2); Compression garments (n = 4); External pneumatic compression (n = 5)	Whole-body cryotherapy (n = 51); Cold-Water immersion (n = 64); Contrast Water Therapy (n = 13)

**Table:** 

			**Number of recovery modalities used after a training day**			
Number of recovery strategies implemented per session	Total		n			
1	17	1	2	0	1	13
2	61	27	19	5	21	49
3	40	32	26	5	23	34
4	21	22	15	6	17	24
5	7	8	8	5	6	8
Total	146	90	70	21	68	128

*The total number of times players chose a specific recovery modality after a training day is reported as n*.

### Mixed Linear Model Results

We did not observe any significant interactions between clusters and recovery modalities (all *p* > 0.05). The linear mixed-model regression analysis revealed that cooling strategies were associated with Δ*muscle soreness* (β = −1.00, 95% confidence interval [CI] [−1.8, −0.1], *p* = 0.02). The LRT confirmed that cooling strategies significantly reduced muscle soreness (χ^2^ = 4.93, *p* = 0.02).

### Comparison of Cold Recovery Modalities

Because we noticed a significant association between cooling strategies and Δ*muscle soreness*, we compared the effect of each modality on the same subjective recovery variables. We observed no significant differences between CWI, CWT and WBC for Δ*muscle soreness* (*p* = 0.33), Δ*perceived fatigue* (*p* = 0.60) or *sleep quality* (*p* = 0.45). Kruskal-Wallis analyses revealed significant differences between cold recovery modalities (*p* = 0.03) for *perceived recovery*, with a higher score for WBC compared with CWI (*p* = 0.02). No significant differences were observed among the other cooling techniques (all *p* = 1.0).

## Discussion

The present study reported 3 major findings: (i) professional tennis players consistently adopt recovery methods after training, primarily utilizing a combination of 2–3 techniques, with cooling techniques being the most widely used modality; (ii) *muscle soreness* and *perceived fatigue* are not significantly different depending on training profile clusters; and (iii) only cooling strategies were found to be efficient for attenuating *muscle soreness*, regardless of the training type cluster.

Given that 91% of considered sessions were monitored between mid-November and the end of December, our data may represent the distribution of training loads during the preseason period. Tennis players daily experienced a total sRPE training load of 1,246 ± 471 A.U. per day, which is similar to the total sRPE training load of 1,267 A.U. that was recently reported by Murphy et al. (2015) for high-performance junior tennis players during a 4-week pre-competition period. In more detail, the daily tennis training volume obtained in our study (130.3 ± 41.0 min) was similar to the 151.0 ± 12.1 min daily training volume reported by Murphy et al. (2015). In contrast, they observed shorter strength and conditioning volumes (45.0 ± 14.9 min), in comparison with the 95.5 ± 50.2 min observed in our study. This observation is consistent coherent with the increase in strength training loads that occur between 17 and 20 years of age, as the players involved in our study were approximately 3 years older, on average, than the players in the Murphy et al. (2015).

Three clusters, representing the different types of classic training days, were identified among the professional tennis players in this study. The “combined tennis and S&C training” cluster better reflects a typical training day, as it represented the majority of monitored sessions (*n* = 97). In addition, the total sRPE training load, tennis training duration, and strength and conditioning training duration values for “combined tennis and S&C training” cluster were similar to the overall means calculated for all sessions. Thus, a typical training day appears to consist of an approximately evenly distributed volume of tennis and strength and conditioning training. Training days with a predominant volume of either tennis (“Tennis-specific oriented training” cluster, *n* = 18) or strength and conditioning (“S&C oriented training” cluster, *n* = 31) training appears to be less experienced by professional tennis players who compete in international tournaments.

Contrary to our hypothesis, the training clusters did not show any significant differences for *muscle soreness* or *perceived fatigue*, either before or after recovery intervention. This finding was not consistent with the findings of previous studies, which showed a positive correlation between training load and *muscle soreness* or *perceived fatigue* in professional football players (Moalla et al., 2016; Thorpe et al., 2017). However, this potential association remains controversial because, to our knowledge, no study has provided any theoretical and validated explanation to support these findings (Saw et al., 2016). Furthermore, *muscle soreness* and *perceived fatigue* can be elevated for up to 72 h following matches or training. Some items may have been more sensitive to differences in the clusters if additional time points had been collected later in the recovery time-course (Ojala and Hakkinen, 2013). In this context, the level of fatigue has recently been reported to be more sensitive to accumulated training days among professional football players (Thorpe et al., 2017). Future research is warranted to explore the impacts of accumulated and chronic training loads on these subjective variables in professional tennis players.

After a day of training, 86.3% of the professional tennis players included in the current study used a combination of 2–5 recovery modalities, with 69.2% of players using 2 or 3 modalities. This observation is in line with a recent study reported by Fleming et al. (2018) which indicated that 80% of competitive tennis players used multiple recovery modalities after a match. A combination of at least 2 recovery modalities appeared to be a well-integrated post-training habit among the professional tennis players involved in this study. The only study that explored the effects of a combined mixed-method recovery intervention found that the combination of 3 recovery modalities CWI, compression garments, and sleep-hygiene recommendations) was effective for reducing *muscle soreness* after twice-a-day, on-court, tennis sessions (Duffield et al., 2014). This finding could, therefore, be considered to be reflective of the progressive transfer of evidence-based knowledge into recovery practices in tennis.

More than 83% of players performed a cooling intervention (CWI, WBC, or CWT) after training, which is supported by similar recent studies, highlighting the considerable use of cooling strategies, particularlyCWI, among elite rugby players (Tavares et al., 2017), professional soccer teams (Nedelec et al., 2013), and competitive tennis players (Fleming et al., 2018). Similar to other sports, passive mobilization (stretching) and low-limb blood flow stimulation (active recovery) were also used frequently by the players included in this study (Bahnert et al., 2013). The easy access to recovery methods available at the Tennis Center may have influenced the athletes' behaviors and biased the selection of recovery routines (Bahnert et al., 2013). Because a substantial number of training sessions (146 sessions) was monitored among a large cohort of players (35 professional players), the present data can confidently be considered to be reflective of the recovery habits adopted by professional tennis players during preparatory/pre-competitive phases in a high-level environment.

Using this representative cohort, mixed linear models allowed us to independently test the impacts of clusters and recovery methods on the subjective variables. First, the absence of significant associations between subjective variables and clusters confirmed that neither *muscle soreness* nor *perceived fatigue* was related to the total sRPE training load or content. Our primary finding was that cooling techniques were significantly associated with attenuated *muscle soreness* (Table 2) the following day (12–16 h after training). These results are consistent with previous research that revealed positive impacts associated with CWI (Nedelec et al., 2013; Ihsan et al., 2016), WBC (Hausswirth et al., 2011), and CWT (Bieuzen et al., 2013) on decreased *muscle soreness*. Similar results were found by Duffield et al. (2014), who reported a significant decrease in *muscle soreness* the morning after a tennis training day when a combination of CWI, compression garments, and sleep education was applied. Recent research reported no positive effects for CWI or WBC on muscle soreness sustained by recreational athletes after a fatiguing protocol performed under controlled laboratory conditions, which could be imperfectly representative of the degree of muscle damage induced by professional tennis practice (Wilson et al., 2018). The common purpose of recovery techniques is to decrease cutaneous, muscle, and core temperatures (Costello et al., 2012; Bieuzen et al., 2013; Ihsan et al., 2016), to induce an analgesic effect during the first hours after exercise, via the inhibition of nociceptors. Such acute mechanisms were unlikely to influence the present measurements, as data regarding muscle soreness and other subjective variables were collected between 12 and 16 h post-recovery (Ihsan et al., 2016). Cooling more likely limited edema formation and inflammatory responses, through the modulation of blood flow (CWI, WBC, and CWT) and the stimulation of fluid transport (CWI and CWT), thereby, decreasing muscle soreness (Costello et al., 2012; Ihsan et al., 2016). We also cannot completely exclude a potential placebo effect, as demonstrated by recent studies (Broatch et al., 2014; Wilson et al., 2018), even if this observation was made in recreational athletes with very different training histories and objectives than those characteristics of professional training players.

**Table 2:** Results of the mixed-effects models testing the distinct effect of training clusters and recovery modalities on subjective variables.

		**Δmuscle soreness**			**Δperceived fatigue**			**Sleep quality**			**Perceived recovery**		
		**β**	***CI***	***p-value***	**β**	***CI***	***p-value***	**β**	***CI***	***p-value***	**β**	***CI***	***p-value***
	**FIXED EFFECT**												
Training clusters	Intercept	0.26	[−0.8; 1.3]	0.61	−0.60	[−1.8; 0.6]	0.32	3.85	[2.6; 5.1]	0.00	5.12	[4.1; 6.2]	0.00
	Cluster 2	−0.45	[−1.4; 0.5]	0.34	−0.52	[−1.6; 0.5]	0.33	0.44	[−0.7; 1.6]	0.42	−0.14	[−1.0; 0.7]	0.74
	Cluster 3	0.31	[-0.4; 1.0]	0.39	−0.15	[−1.0; 0.6]	0.71	0.29	[−0.5; 1.1]	0.49	−0.01	[−0.7; 0.6]	0.96
Recovery modalities categories	Cooling strategies	−1.00	[−1.8; −0.1]	0.02^*^	−0.48	[−1.5; 0.5]	0.33	−0.32	[−1.4; 0.7]	0.54	0.12	[−0.7; 1.0]	0.78
	Heating strategies	−0.17	[−1.0; 0.7]	0.70	−0.11	[−1.1; 0.9]	0.83	0.04	[−1.0; 1.1]	0.93	0.65	[−0.2; 1.5]	0.14
	Passive mobilization	−0.11	[−0.8; 0.5]	0.72	−0.04	[−0.8; 0.7]	0.91	−0.23	[−1.0; 0.5]	0.53	0.44	[−0.2; 1.1]	0.15
	Lower limb blood flow stimulation	−0.04	[−1.0; 0.3]	0.25	−0.26	[−1.0; 0.4]	0.48	0.06	[−0.7; 0.8]	0.87	−0.27	[−0.9; 0.3]	0.36
	Physiotherapy techniques	−0.17	[−0.8; 0.4]	0.59	0.43	[−0.3; 1.1]	0.24	0.14	[−0.6; 0.9]	0.70	0.16	[−0.4; 0.8]	0.59

*β: small point estimates; CI: 95% confidence intervals*;

* *p < 0.05;* Δ *muscle soreness = muscle soreness (POST)—muscle soreness (PRE);* Δ *perceived fatigue = perceived fatigue (POST) perceived fatigue (PRE)*.

Contrary to the results of our study, 20–30 min of massage performed after exercise has been reported to reduce delayed-onset muscle soreness for up to 72 h post-exercise (Guo et al., 2017), as confirmed by a recent meta-analysis that found that massage was the most effective technique for reducing muscle soreness (Dupuy et al., 2018). Similarly, foam rolling (Wiewelhove et al., 2019), electrostimulation (Borne et al., 2015), and compression garments (Marqués-Jiménez et al., 2015) have all been demonstrated to attenuate muscle soreness after exercise. Exercise may induce various physiological and psychological stresses, depending on numerous factors, such as mode, duration, or training status (Halson et al., 2014). However, some previous studies (Guo et al., 2017; Dupuy et al., 2018; Wiewelhove et al., 2019) have been meta-analyses, combining data from multiple various fatiguing protocols, which have very limited transfer to ecological contexts due to the lack of distinction in the levels of muscle soreness induced by exercise and the training levels of the athletes. The present study appraised real and ecological psychophysiological responses to training and recovery in professional tennis players. The potential positive effects of certain recovery interventions (foam rolling and electrostimulation), which are less commonly utilized by tennis players, may have been concealed by the use of more popular recovery techniques that have been demonstrated to be inefficient in the literature (stretching and active recovery) (Van Hooren and Peake, 2018). Although cooling strategies significantly alleviated muscle soreness, none of the cold recovery modalities that were monitored in the present study attenuated *perceived fatigue*, in contrast with the positive effects of cold modalities and/or massages that have previously been reported in the literature (Dupuy et al., 2018). Contrary to previous studies performed in elite athletes, we did not observe improved *sleep quality* following WBC and CWI (Schaal et al., 2015). However, these reports were anecdotal, and most research studies have found little evidence for improved sleep after cold application (Broatch et al., 2019).

Because the underlying mechanisms may differ between different cold techniques, we strived to distinguish the respective effects of each cooling strategy used by professional players. No significant differences between cold modalities were observed for *muscle soreness* of *perceived fatigue*. Based on previous studies, we expected a greater decrease in *muscle soreness* after CWI or CWT compared with WBC (Bleakley et al., 2012; Hohenauer et al., 2015). WBC showed a significant increase in *perceived recovery* compared with CWI. These results are in line with a study reported by Hausswirth et al. (2011) (+21.7 on a 100-point rating scale), who showed an increase in *perceived recovery* after WBC compared with passive condition 24 h after a simulated trail run. Indeed, previous studies have revealed that WBC may increase norepinephrine and dopamine release, resulting in an additional analgesic effect and the increases perception of recovery and well-being. Inversely, a previous study showed no effect of CWI on psychological recovery after exercise (Cheung et al., 2003). However, considering the time-course of subjective variable measurements (> 12 h post-recovery), these findings should be considered with caution, as the timing of norepinephrine and dopamine release in response to cooling strategies remains unclear. These latter statistical comparisons between cold modalities were different from the mixed linear model because they did not independently test the effects of each intervention. Other techniques used in combination with a cooling strategy could, therefore, influence subjective variables. The high variability in the different subjective variables confirmed that responses to recovery interventions are specific and individual.

## Limitations

Some methodological considerations should be noted when interpreting the present absolute values of subjective recovery variables. First, training clusters may have elicited significantly different effects on subjective recovery variables if no recovery interventions (i.e., a control condition) had been implemented. However, this condition would not be representative of real-world professional athlete conditions. We used the linear mixed model to overcome this bias, by estimating each subjective recovery variable for each training cluster while excluding the potential effects of recovery modalities. Second, raw data of subjective variables (Δ*muscle soreness*, Δ*perceived fatigue*, sleep quality, and perceived recovery) were unfortunately not available as players mostly used a combination of modalities. Thus, linear mixed model allowed to estimate subjective variables that would likely be recorded for each recovery modalities categories (Table 2). Third, one should acknowledge that some key variables recognize to alter subjective recovery were not controlled in the present study, such as menstrual cycle or travel. However, the data collection period, which was restricted to the training phase, limited the potential influences of travel or jet lag on fatigue. Fourth, the data collection period was circumscribed to the training phases of players (≈ 40% of the season). On the one hand, this controlled period of time restricted the number of training sessions that could be monitored for each player. On the other hand, it limited the potential influences of travel or jet lag on fatigue.

## Conclusion

This study showed that professional tennis players face substantial daily training loads (total sRPE training load) during training periods, with no consistent impacts on acute subjective recovery. Future research should investigate the potential impacts of accumulated training loads over longer periods of time. The benefits of recovery routines consisting of multiple recovery techniques appear to be well-anchored in practice. During general, specific preparations or during the taper period, cold modalities appear to efficiently decrease tennis training-induced muscle soreness compared with other recovery techniques. However, future research should include more data, with homogeneous repartition between recovery interventions, to compare the efficiencies of different combinations of recovery interventions. Although effective, cold recovery should be implemented at key strategic moments, to limit fatigue without blunting expected adaptations. The inter-individual variability observed among the perceived responses to training loads and recovery strengthens the necessity to perform continuous training load monitoring to improve recovery periodization, based on individual training-induced fatigue.

## Data Availability Statement

The raw data supporting the conclusions of this article will be made available by the authors, without undue reservation.

## Ethics Statement

The studies involving human participants were reviewed and approved by Committee of Sud Méditerranée IV (no 17 10 05). Written informed consent to participate in this study was provided by the participants' legal guardian/next of kin.

## Author Contributions

MP, FB, BM conceived and designed research. MP conducted experiments. MP, FB, GG, QL, BM analyzed data. MP, FB, GG wrote the manuscript. All authors contributed to the article and approved the submitted version.

## Conflict of Interest

The authors declare that the research was conducted in the absence of any commercial or financial relationships that could be construed as a potential conflict of interest.
