# Supplementary material for: The Impact of Recovery Practices Adopted by Professional Tennis Players on Fatigue Markers According to Training Type Clusters
Source: Front Sports Act Living. 2020 Sep 2;2:109. doi: 10.3389/fspor.2020.00109 (PMC7739815; doi:10.3389/fspor.2020.00109)
Supplement: Supplementary file 1 [file Table_1.DOCX]

|  | **Combined and S&C training cluster**  **(n=97)** | | | | **Tennis specific oriented cluster**  **(n=18)** | | | | **S&C oriented cluster**  **(n=31)** | | | |
| --- | --- | --- | --- | --- | --- | --- | --- | --- | --- | --- | --- | --- |
|  | Subjective variables (A.U) | | | | | | | | | | | |
|  | **J-0 (PRE)** | | | | | | | | | | | |
|  | Mean |  | SD | Range | Mean |  | SD | Range | Mean |  | SD | Range |
| Muscle soreness | 4.1 | ± | 2.2 | 0 - 8.6 | 5.2 | ± | 2.3 | 0.1 - 8.3 | 4.7 | ± | 2.1 | 4.8 - 8.7 |
| Perceived fatigue | 5.0 | ± | 2.1 | 0.6 - 10 | 6.0 | ± | 1.8 | 1.9 - 8.5 | 5.7 | ± | 2.2 | 1.3 - 9.3 |
|  | **J+1 (POST)** | | | | | | | | | | | |
|  | Mean |  | SD | Range | Mean |  | SD | Range | Mean |  | SD | Range |
| Muscle soreness | 3,3 | ± | 2,0 | 0,0 – 7.7 | 4.0 | ± | 2.3 | 0.0 – 8.1 | 4.1 | ± | 1.9 | 0.4 - 8.0 |
| Perceived fatigue | 3.9 | ± | 2.2 | 0.6 – 10.0 | 4.8 | ± | 1.9 | 1.2 – 8.5 | 4.4 | ± | 2.1 | 0.7 - 8.4 |
| Sleep quality | 3.3 | ± | 2.2 | 0 - 8.4 | 4.3 | ± | 2.3 | 0 - 8.5 | 3.9 | ± | 2.1 | 0.1 - 7.6 |
| Perceived recovery | 5.6 | ± | 1.9 | 1.3 - 10 | 4.8 | ± | 1.4 | 2.3 - 7 | 5.5 | ± | 1.6 | 2.4 - 8.8 |

**Table S1. Subjective variables on J-0 (Pre-recovery) and on J+1 (Post-recovery)**

**J-0 (PRE):** After the day training before recovery; **J+1 (POST):** Next morning before training.
